# P-1464. Nasopharyngeal Colonization in South Texas Children less than 6 Years of Age with or without Lower Respiratory Tract Infections

**DOI:** 10.1093/ofid/ofaf695.1650

**Published:** 2026-01-11

**Authors:** Kristina G Hulten, Andrea T Cruz, Lauren M Sommer, Meghan Walther, Linda Lamberth, Victor M Gonzalez, Lesby Mayorquin, Andrea Forbes, Isaac Chavez, Lindsay Grant, Adriano Arguedas, Maria J Tort, Ashley Miller, Cody Bender, Alejandro D Cane, Bradford Gessner, Sheldon L Kaplan

**Affiliations:** Baylor College of Medicine, Houston, TX; Baylor College of Medicine, Houston, TX; Baylor College of Medicine, Houston, TX; Baylor College of Medicine, Houston, TX; Baylor College of Medicine, Houston, TX; Baylor College of Medicine, Houston, TX; Baylor College of Medicine, Houston, TX; Baylor College of Medicine, Houston, TX; Baylor College of Medicine / Texas Children's Hospital, Houston, Texas; Pfizer Inc., Collegeville, PA; Pfizer, Collegeville, Pennsylvania; Pfizer, Inc, Collegeville, Pennsylvania; Pfizer, Collegeville, Pennsylvania; Pfizer Vaccines, Collegeville, Pennsylvania; Pfizer, Collegeville, Pennsylvania; Pfizer, Inc., New York, New York; Baylor College of Medicine, Houston, TX

## Abstract

**Background:**

Recently, two new pneumococcal conjugate vaccines (PCV15 and PCV20) were introduced in the United States (US). We sought to define the current nasopharyngeal (NP) colonization prevalence and serotype distribution of pneumococci (SPN) obtained from children seeking care at Texas Children’s Hospital, Houston, TX, US, for respiratory infections and to report their PCV status.

**Table 1.**
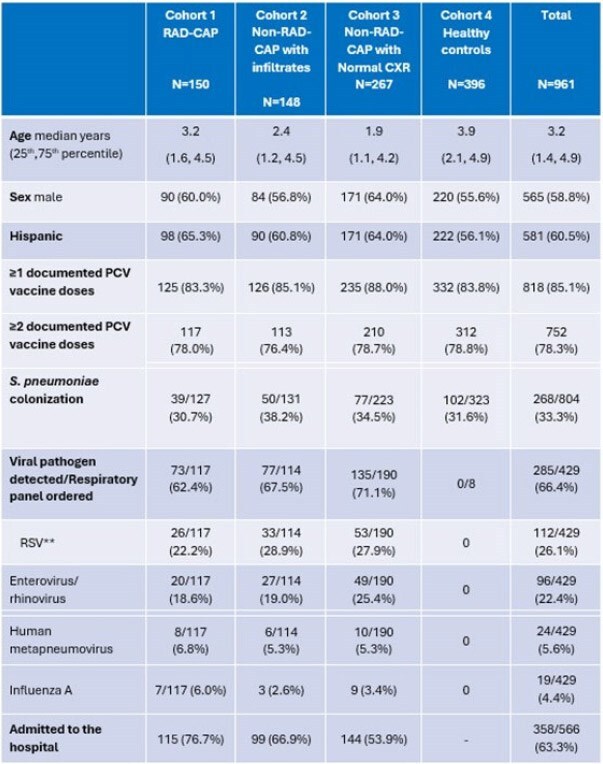
Demographic and clinical characteristics of the study population * 143 children were either recorded as unvaccinated (n=12), or their parents expressed uncertainty regarding vaccination status and their PCV vaccine records were not available. **Respiratory syncytial virus

**Figure 1.**
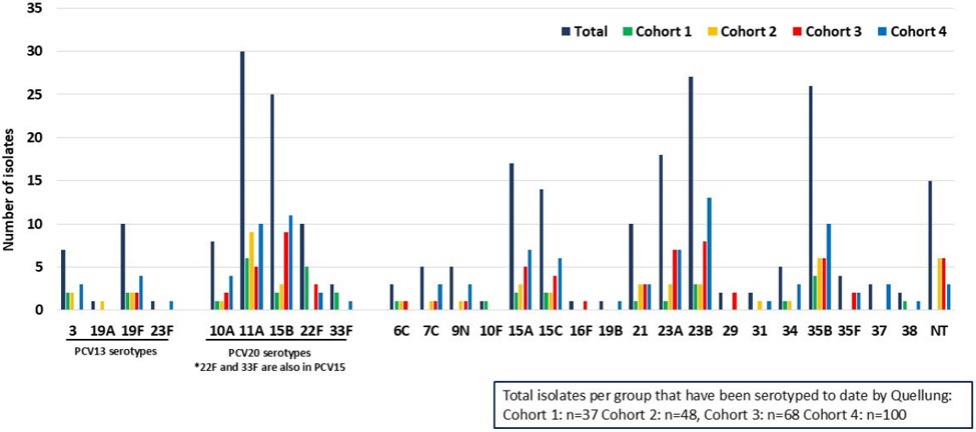
Serotype distribution of nasopharyngeal isolates from children <6 years old, 2023-2025

**Methods:**

NP specimens were obtained as part of an ongoing 2-year study on children 3 months – 6 years old who were enrolled into four distinct cohorts: Radiologically confirmed community acquired pneumonia (RAD-CAP) (1), non-RAD-CAP respiratory infections with (2) or without (3) CXR findings, and healthy controls (4). Medical history and laboratory test results performed as part of standard care were obtained. PCV status was obtained from medical records or a state registry. Children with certain underlying conditions were excluded from the study. Specimens from April 2023- March 2025 were included. NP swabs were inoculated in STGG media and stored frozen at -80°C. Cultures and antibiotic susceptibilities were performed by standard methods. Serotypes were determined by Quellung reaction.

**Results:**

Of 961 children enrolled, 915 (95.2%) had a swab collected. To date, 839 swabs have been cultured of which 268 (31.9%) grew SPN, equally across cohorts. (Table). The most common serotypes were 11A (11.9%), 23B (10.7%), 35B (10.3%), and 15B (9.9%). Nineteen isolates were PCV13 serotypes, most commonly 19F. (Figure) One isolate (serotype 23F) had a penicillin MIC >2 µg/ml (i.e. non-susceptible by Clinical Laboratory and Standards Institute (CLSI) guidelines for IV treatment of non-CNS SPN infections). RSV was the most common respiratory virus identified. Among 818 children with vaccination data and at least one dose of PCV, 724 were vaccinated with one or more doses of PCV13, 42 (5.1%) had received at least one dose of PCV15, 152 (18.6%) had received at least one dose of PCV20. Twelve were reported unvaccinated.

**Conclusion:**

Initial data indicate serotypes 11A and 15B (both contained in PCV20, as well as 23B and 35B (not contained in any licensed pediatric PCV) are common colonizers among children age < 6 years old in Houston. All but one isolate was penicillin susceptible for treating non-CNS infections.

**Disclosures:**

Kristina G. Hulten, PhD, Pfizer: Grant/Research Support Andrea T. Cruz, MD, MPH, Pfizer: Grant/Research Support Lindsay Grant, PhD, MPH, Pfizer: Employee|Pfizer: Stocks/Bonds (Private Company) Adriano Arguedas, Medical director, Pfizer employee: employee|Pfizer employee: Stocks/Bonds (Public Company) Maria J. Tort, PhD, Pfizer, Inc: Stocks/Bonds (Public Company) Ashley Miller, MA, CCRP, Pfizer: Stocks/Bonds (Public Company) Alejandro D. Cane, MD, PhD, Pfizer Inc.: All authors are employees of Pfizer Inc. and may hold stock and/or stock options of Pfizer Inc. Sheldon L. Kaplan, MD, Pfizer: Grant/Research Support

